# Evaluation of a Required Vertical Point-of-Care Ultrasound Curriculum for Undergraduate Medical Students

**DOI:** 10.7759/cureus.30002

**Published:** 2022-10-06

**Authors:** Zachary Boivin, Sandra Carpenter, Grace Lee, Brock Chimileski, John Harrison, Dharamainder Choudhary, Meghan Herbst

**Affiliations:** 1 Emergency Medicine, University of Connecticut Emergency Medicine Residency, Farmington, USA; 2 General Surgery, Beth Israel Deaconess Medical Center, Harvard Medical School, Boston, USA; 3 Medicine, University of Connecticut School of Medicine, Farmington, USA; 4 Emergency Department, Yale New Haven Hospital, New Haven, USA; 5 Orthodontics, University of Connecticut School of Medicine, Farmington, USA; 6 Surgery, University of Connecticut School of Medicine, Farmington, USA; 7 Emergency Department, University of Connecticut School of Medicine, Farmington, USA

**Keywords:** point-of-care ultrasonography, handheld ultrasound, medical school education, ultrasound (u/s), medical school ultrasound curriculum

## Abstract

Introduction

Point-of-care ultrasound training beginning in undergraduate medical education reinforces anatomy and physical examination skills and enhances clinical care. Implementation in an overcrowded curriculum requires strategic planning to overcome barriers including lack of faculty and equipment. Using Kern’s six-step model as a framework, our study question was whether a longitudinal point-of-care ultrasound curriculum threaded through four years of medical school and using a novel combination of evidence-based strategies was feasible, acceptable, and resulted in students achieving ultrasound competencies by graduation.

Materials and methods

From 2016 to 2020, a required, vertical point-of-care ultrasound curriculum was created across all four undergraduate medical education class years, spearheaded by a single ultrasound fellowship-trained emergency physician with support from two basic anatomy faculty. We utilized strategies including handheld ultrasound devices, near-peer teaching, flipped classroom with virtual learning modules, staggered station rotations, and gamification to optimize student-instructor ratios and faculty time. Surveys and timed objective structured clinical assessments evaluated the curriculum.

Results

Students from the class of 2022 (n=99, 100% of class) participated in all curricular elements. Senior students answered more survey knowledge questions correctly when compared to pretest questions answered by first- and second-year students. Among 84 students who completed the survey, 75 (89%) rated their ultrasound curriculum as superior or above average. Objective structured clinical examination scores recorded for 53 students (54% of the class) demonstrated students correctly identified a median of 11-18 structures (interquartile range: 9.5-13) using point-of-care ultrasound.

Conclusion

Evidence-based strategies allowed faculty to develop a four-year required ultrasound curriculum that was highly acceptable by students and improved their knowledge and skills at graduation. At low cost and with few faculty, this program has been sustained for over six years.

## Introduction

Point-of-care ultrasound (PoCUS) has been shown to decrease medical errors, provide efficient real-time diagnosis, and augment other imaging modalities [1,2]. PoCUS also complements anatomy, reinforces physical examination skills, facilitates bedside diagnosis and management, and is therefore a valuable learning tool for physicians in training [3]. For these reasons, training in PoCUS has become a highly desirable component of both graduate and undergraduate medical education (UME), to prepare physicians of all specialties.

Recently published reviews of longitudinal ultrasound curricula at the UME level demonstrate considerable heterogeneity among institutions, partly due to a current lack of national standards and guidelines for PoCUS training in UME [4-7]. However, a principal limitation is a resource-intensive foundation for a PoCUS curriculum, with commonly cited barriers being costs of ultrasound machines, recruitment of trained instructors, hiring of patient models, and insufficient time in the curriculum [4,6-7].

The aim of this study was to determine whether a mandatory longitudinal PoCUS curriculum threaded through four years of medical school and using a novel combination of evidence-based strategies was feasible, acceptable, and resulted in students achieving ultrasound competencies by graduation. There is currently no published four-year PoCUS curriculum that describes both the use of handheld ultrasound devices and near-peer teaching to increase hands-on time for students and decrease institutional cost.

## Materials and methods

This was a prospective observational study that used historical controls as the comparison group. The University of Connecticut School of Medicine Institutional Review Board (IRB) deemed this project exempt, as a quality improvement project (#SM1220). We sought to assess the stakeholders, start-up, maintenance, and outcomes of creating a vertical PoCUS UME curriculum at our institution with goals of maximizing hands-on learning and minimizing costs. All students enrolled at the time of curriculum development participated in PoCUS sessions. We used Kern’s six-step model as a framework to describe our approach to curriculum development [8].

Step 1: identify the problem and perform a general needs assessment

For classes graduating prior to 2020, there was no PoCUS curriculum at our institution, a public four-year university-based medical school with several affiliated community medical centers, which graduates approximately 102 students annually. A survey of prior graduating classes demonstrated that over 90% of students preferred more UME PoCUS training, which is consistent with surveys performed at other institutions [7,9]. Furthermore, the widespread utilization of PoCUS education, regardless of specialty, made the need for incorporating education at the UME level evident [9,10]. The hands-on ultrasound program was initiated for first-year medical and dental students in 2016. Overall restructuring of the four-year undergraduate medical curriculum at this time provided an ideal opportunity to gradually integrate the new vertical PoCUS curriculum one year at a time, with all components in place by 2020.

Our ideal teaching approach centered around small group learning and hands-on scanning, which can be challenging with limited faculty and resources [4,11]. An emergency physician with ultrasound fellowship training was hired in 2017 with 0.25 full-time equivalent (FTE) to update and direct third- and fourth-year medical education as Clinical Clerkships Director, who spent approximately 150 h developing the curriculum and 82 h annually maintaining it. Two basic sciences faculty dedicate approximately 60 h annually to ultrasound sessions across years one and two as part of the anatomy curriculum. Equipment purchased by the medical school to implement this curriculum included four SonoSim Ultrasound Training Solutions (SonoSim, Inc.: Santa Monica, CA) that provide interactive simulated ultrasound cases, two cart-based ultrasound machines (SonoSite M-Turbo; FUJIFILM Sonosite: Bothell, WA), 40 handheld ultrasound units with unlimited cloud storage (Butterfly iQ; Butterfly Network: Guilford, CT), and 20 tablets (Apple iPads; Apple Inc.: Cupertino, CA) at a total cost of approximately $216,000, plus $100 annually for the unlimited cloud storage.

Step 2: targeted needs assessment

A sample of 18 fourth-year students from the graduating class of 2019, who had not experienced the PoCUS curriculum and were enrolled in a residency preparation elective, took a brief survey to establish student baseline PoCUS knowledge. All students enrolled in this elective participated in the survey. The average score was 26%, demonstrating a general lack of knowledge about ultrasound orientation, technique, sonoanatomy, sonopathology, and basic ultrasound physics essential for PoCUS performance and effective use. Eighty-five percent reported either no exposure to or difficulty with acquiring appropriate views of the aorta, heart, inferior vena cava, focused assessment with sonography in trauma (FAST), kidneys, lung, and peripheral intravenous access. Seventy-one percent reported they had no exposure to or were not able to interpret views of the same examinations. All but one student believed their medical school ultrasound curriculum was below average or poor on a five-point Likert scale, and one student reported that it was average, demonstrating a need for a formal ultrasound curriculum.

Step 3: goals and objectives

From the needs, assessments, and faculty discussions, we created the goals for the vertical PoCUS curriculum for graduate medical students with a strong ultrasound anatomy foundation, confidence in their skills, and interest in appropriate PoCUS utilization during residency. The objectives of the curriculum are - (1) to explain the basic physics and knobology of ultrasound, (2) to recognize appropriate indications for PoCUS, (3) to acquire ultrasound images across organ systems using multiple devices, (4) to interpret and differentiate between sonoanatomy and sonopathology, and (5) to incorporate PoCUS findings to inform clinical decision-making. Specific learning objectives of PoCUS sessions are carefully aligned with objectives of other courses taking place at each stage of the students’ UME curriculum.

Steps 4 and 5: educational strategies and implementation

PoCUS instruction starts with ultrasound anatomy correlation for first-year medical students, within the preclinical course that includes human anatomy. Second-year students learn how to integrate PoCUS with physical examination findings in the physical diagnosis course. Third- and fourth-year students practice PoCUS skills in an orientation week just prior to clinical clerkships, fall mid-clerkship week, spring week following the last third-year clerkship, and a required Transition to Residency two-week course at the end of year four. Hands-on sessions are typically 1-2 h and are taught in small student groups of two to five students, intermittently over a one-week period. Instructors use the Socratic method to teach and actively connect new knowledge to existing knowledge. Pre- and post-knowledge tests for students are administered at each session to reinforce key learning points. Approximately 37 h of in-person PoCUS training are integrated with pre-existing course learning objectives over the four years of medical school (Table 1).

**Table 1:** Summary of point-of-care ultrasound session organization across UME curriculum. PoCUS: point-of-care ultrasound; UME: undergraduate medical education

UME year	Realm of PoCUS	Instruction	Hours
Year 1	Anatomy correlation	Faculty	16
Year 2	Anatomy correlation physical examination correlation	Faculty near-peers	8
Year 3	Clinical vignette-based correlation	Near-peers	10
Year 4	Vascular access ultrasound algorithms specialty-specific breakout sessions	Near-peers and near-peers+faculty	3

All PoCUS sessions are required as part of larger courses; these courses are graded pass/fail based on successful completion of all elements. The following strategies were incorporated to implement a required four-year vertical UME PoCUS curriculum with effective small group hands-on learning without imposing excessive burden on faculty.

Handheld Ultrasound Devices

Handheld ultrasound devices are popular for their versatility, portability, and accessibility and are particularly useful in medical education [12,13]. Handheld ultrasound devices incorporated into UME improve student understanding of anatomical concepts and enhance student confidence and diagnostic accuracy in the physical examination [13].

Our institution purchased 40 handheld ultrasound devices (Butterfly iQ) with 20 tablets (Apple iPad) in place of one expensive cart-based machine. All handheld ultrasound devices are securely maintained in our simulation center. They can be readily transported on a single cart to any on-campus location for in-class instruction or temporarily loaned individually to students for independent practice. Hands-on PoCUS sessions consist of small groups of two to five students, with at least two handheld ultrasound devices per group. Students rotate between performing ultrasound under supervision of a trained instructor, observing peers, and serving as patient models. Students may use the handheld ultrasound devices and upload their acquired images into a shared cloudspace. Uploaded images can be promptly reviewed by ultrasound faculty who provide feedback to students.

Near-Peer Teaching

The recruitment of experienced instructors is a consistent barrier to implementing longitudinal PoCUS curricula [4,7]. Near-peer teaching allows for small student-to-instructor ratios without overextending faculty time or hiring additional faculty. Near peers are comparable to traditional faculty in early skill development and may also increase learner comfort [11,14,15]. For the instructors, near-peer teaching improves academic performance and confidence in the skill being taught [16].

We recruit third- and fourth-year medical students and residents who have completed formal ultrasound coursework and training as near-peer instructors. Students are eligible if they have completed a minimum of a two-week emergency medicine-based ultrasound elective. Residents are eligible if they have completed a minimum of 16 hours of PoCUS formal coursework and 100 logged PoCUS examinations. Resident near-peer instructors perform an average of 550 diverse PoCUS examinations prior to teaching in the UME course. We accommodate existing schedules and provide a small incentive, such as a $5 gift card. Near-peer instructors are provided with a faculty guide and ample time to review the guide prior to the session. Key images of sonopathology are highlighted in the faculty guide and are accessible for demonstration, during teaching, through the website image library. The lead ultrasound faculty member is available at each session to supervise and troubleshoot as needed. The third- and fourth-year course elements are led primarily by fourth-year student and resident near-peer instructors, respectively.

As an example of near-peer teaching in action, third-year medical students attend a 90-minute session where they perform PoCUS at three different stations to determine the etiology of undifferentiated hypotension. Students are divided into groups with a student-to-instructor ratio of 2:1 and scan a live model using handheld ultrasound devices under direct supervision by a near-peer teacher. The examination is informed by a vignette describing the patient presentation. Students receive real-time feedback from the near-peer teacher as they scan, and clips of the pertinent sonopathology are displayed via mobile device once the appropriate technique and sonoanatomy are demonstrated. Students then incorporate PoCUS findings to generate a list of possible diagnoses and propose a treatment plan. Near-peer teachers rotate through the student groups so that students are exposed to multiple clinical vignettes and instructors throughout the session.

Flipped Classroom and Virtual Learning Modules

The flipped classroom model, involving remote student learning before in-classroom instruction, can reduce faculty time, cost, and resources, and also tailors learning to the needs of the individual student [17,18]. Virtual learning environments may promote student engagement, satisfaction, and information recall [19,20].

Prior to each PoCUS session, students are asked to watch a brief video created by ultrasound faculty. We created a narrated ultrasound video series that complements live ultrasound sessions and covers sonoanatomy and sonopathology, anatomical planes and orientation, technique, clinical integration, and ultrasound-guided procedures. The series, consisting of 12 videos ranging from 8 to 20 minutes, is intended to be viewed in sequential order, but no prerequisite knowledge is needed by learners before the introductory video. This narrated series is student-focused, shareable, brief, and animated. Links for the videos are also disseminated to near-peer and faculty instructors to ensure alignment of learning objectives and teaching. A PoCUS website was subsequently developed to house all narrated videos, an image library, and commonly cited ultrasound publications [21].

Small Group Station Rotations/Staggered Station Rotations

Small group rotations create environments for effective skill learning, ideally with a student-to-instructor ratio below 4:1 [11,22]. In order to incorporate PoCUS teaching to a large class of over 100 students, students are divided into three or four smaller groups of 25-35 students, staggered across a curricular day or across a curricular week. Other, non-PoCUS sessions are scheduled at the same time so that students rotate through all sessions and faculty can accommodate an entire class in small groups over the course of the day or week. An example of this is during the mid-clerkship week when an entire class of 110 students is on campus to learn clinical topics not housed in a particular clerkship rotation (see Table 2 for sample schedule).

**Table 2:** Sample group station rotation schedule integrating point-of-care ultrasound sessions. PoCUS: point-of-care ultrasound

Scheduled times	Simulation (three groups of nine students)	Journal club (three groups of nine students)	Clinical Skills (10 groups of two to three students)	PoCUS (10 groups of two to three students)
9:15-10:45	A	B	C	D
11:00-12:30	D	A	B	C
1:15-2:45	C	D	A	B
3:00-4:30	B	C	D	A

Another example takes place during fourth-year students’ Transition to Residency course, where three hours are dedicated to PoCUS. Students rotate through an ultrasound-guided vascular access skill lab, a “free scan” practice station, and a multi-specialty room. In the multi-specialty room, different stations are led by residents or faculty, from different medical specialties, to discuss how they use ultrasound in their practices. Students are pulled from the free scan room one at a time for an objective structured clinical examination (OSCE) with the lead ultrasound faculty member. OSCEs are time-intensive; the presence of simultaneous skill-based small group learning sessions in close proximity allows for efficient time use. Additionally, faculty running OSCEs are also available, for questions or assistance, for the vascular access lab or the free scan room.

Gamification

Gamification is an educational approach that involves the incorporation of game elements into the learning environment, such as teamwork, scoring systems, peer competition, and incentives [23]. We gamify our comprehensive anatomy ultrasound review session at the end of year two by dividing students into teams of five to acquire and discuss PoCUS findings according to presenting symptoms. Points are awarded to the team that best accomplishes this task. A faculty debrief follows each case.

Third-year medical students participate in a one-hour interactive matching exercise called “correlation stations” during their pre-clerkship preparation week. In this exercise, students pair off and match 26 PoCUS images with the corresponding radiograph, CT scan, electrocardiogram, or physical examination finding. For instance, a PoCUS clip of a large pericardial effusion would match with an electrocardiogram demonstrating electrical alternans. Students submit their answer sheets before the end of the activity via Google Forms, and the scores are auto-populated into an Excel spreadsheet. The exercise is followed by a large-group interactive debrief and presentation of a small prize to the winning duo. In this example, gamification enables one faculty member to engage with a large group of students while simultaneously fostering enthusiasm for learning and collaboration between students.

A school-wide ultrasound competition is hosted by the student-led ultrasound interest group in the spring of each year. Students of all years are invited to compete in crossword puzzles, ping-pong tosses, and scan-offs that span PoCUS image-based questions, physics, and foundational knowledge questions. Sixty students competed in 2022.

Extracurricular PoCUS Exposure

Extracurricular engagement is an effective strategy to supplement the barrier of insufficient curricular time. Preclinical students are recruited to serve as volunteer patient models for third- and fourth-year PoCUS sessions. Third- and fourth-year medical students are recruited to serve as near-peer teachers for more junior medical students. A one-week PoCUS elective is offered to up to 20 second-year medical students by lottery and offers an in-depth experience of scanning and incorporating findings into clinical care. Flexible, one- to four-week elective PoCUS experiences are available to third- and fourth-year medical students.

Students of all class years may join events hosted by our student-led ultrasound interest group, which is advised by an ultrasound fellowship-trained emergency physician. This group hosts faculty of all medical disciplines to present cases, practice scanning, and discuss the application of PoCUS in various clinical environments.

Step 6: student assessment, program evaluation, and feedback

The PoCUS curriculum was evaluated through student surveys of knowledge, satisfaction, experiences, and direct observation of hands-on PoCUS skills. All students enrolled at the time of the PoCUS curriculum development participated in the curriculum and associated surveys. No overt exclusion criteria were applied, however, students who were absent during scheduled sessions were not required to reschedule what they missed. The survey instruments were developed by content experts and did not undergo additional testing before implementation.

Comparison of Class Year Knowledge Assessments

Knowledge assessments were conducted via web-based SurveyMonkey (Momentive: San Mateo, CA) surveys. Among a broad mix of questions asked collectively over four years, 12 identical questions were asked of all students at least twice across the vertical curriculum. For each question, the proportion of students who answered the question correctly on first- or second-year pre-tests was compared to the proportion of students who answered the same question correctly on a fourth-year follow-up knowledge assessment. All comparisons were made using N-1 chi-squared test for comparing proportions in MedCalc (MedCalc Software Ltd: Ostend, Belgium) [24]. A Bonferroni correction was applied to account for the 12 comparisons made, and a p-value <0.004 was considered statistically significant. Students had access and were encouraged to view pre-session web-based narrated videos at the time all knowledge questionnaires were distributed.

Self-Assessment of Knowledge, Satisfaction, and Experiences 

In the spring of each year, all graduating fourth-year students were asked to complete a survey consisting of 10 questions designed to assess their perception of the PoCUS curriculum and how they anticipate they would use PoCUS after medical school. We used the survey results from the class of 2022 to evaluate the efficacy and acceptability of the PoCUS curriculum, given that some students in the prior graduating classes missed components of the PoCUS curriculum. The survey was distributed and linked according to student email addresses, without identifying questions. The administering faculty had no influence over the students’ grades or academic performance.

OSCE Assessment of Hands-On PoCUS Skills 

In 2022, graduating fourth-year students enrolled in two of the three two-week Transition to Residency courses (two-thirds of all graduating fourth-year medical students, randomly assigned) participated in an 18-item timed OSCE as part of their final PoCUS session. The OSCE used was modified from others previously published and referenced structures taught across the UME curriculum [23]. Students were given 8 minutes to find 18 anatomical structures with PoCUS (Appendices). All OSCEs were administered by the same ultrasound faculty member.

## Results

A total of 439 students were surveyed and tested at different times in 2021-2022 academic year - 110 first-year, 118 second-year, 112 third-year, and 99 fourth-year students. Of 228 first- and second-year students receiving pre-test questions for the first time, 182 (80%) completed these pre-tests. Of the 329 second- through fourth-year students receiving follow-up knowledge questions (disassociated from teaching sessions), 195 (59%) completed them. Table 3 summarizes the proportions of students across each year answering pre-test and follow-up knowledge questions correctly. First- or second-year students were not asked questions 7 and 12 previously in the form of a pre-test; therefore, proportions correct for the second-year follow-up knowledge assessment were compared with the proportions correct for fourth- and third-year follow-up assessments, respectively, for these questions. The proportions of students answering knowledge questions correctly from pre-test to fourth-year student responses increased for seven of 12 questions (p<0.004). By year four, nearly three-quarters of students answered eight of 12 knowledge questions correctly, compared to only one question with a comparably high proportion of correct answers (70%) for first-year students.

**Table 3:** Student knowledge pre- and post-test PoCUS sessions: proportion of students answering questions correctly by class year. Questions 5-6: 94 of 99 fourth-year students responded. *Questions 7 and 12 were not asked previously of second-year medical students in the form of a pretest, so the second-year follow-up knowledge assessment was compared to fourth- and third-year follow-up assessments, respectively. PoCUS: point-of-care ultrasound

Question	Pre-test first-year (n=105)	Pre-test second-year (n=77)	Second-year (n=70)	Third-year (n=64)	Fourth-year (n=59)*	Pre-test vs fourth-year p-value
1. Which of the following is anechoic on ultrasound?	0.37	–	0.51	0.64	0.63	0.0014
2. Which is the best transducer to evaluate superficial tendons of the wrist?	0.37	–	0.76	0.75	0.68	0.0001
3. What does the top of the screen represent anatomically?	0.31	–	0.79	0.84	0.81	0.0001
4. What tissue appears hyperechoic with posterior acoustic shadowing on ultrasound?	0.61	–	0.96	0.89	0.88	0.0003
5. Which mode is able to detect the directionality of blood flow?	0.48	–	0.92	0.94	0.95	<0.0001
6. Using a lower frequency transducer enables:	0.57	–	0.80	0.80	0.72	0.0281
7. Which is the best transducer to evaluate for a pericardial effusion?	–	–	0.35*	0.38	0.64	0.0011
8. In the setting of a ureteral stone, dilation of which of the following structures is most likely to be visualized on ultrasound?	–	0.66	0.67	0.73	0.73	0.3833
9. Which ultrasound finding is highly specific for a pneumothorax?	–	0.62	0.95	0.9	0.81	0.0167
10. In the subxiphoid view of the heart, the structure seen at the top of the screen is the:	–	0.70	0.89	0.74	0.76	0.4385
11. In which cardiac view does the left ventricle have the most circular appearance?	–	0.56	0.86	–	0.80	0.0034
12. When performing point-of-care biliary ultrasound, which structure is the best landmark for locating the gallbladder?	–	–	0.89*	0.86	–	0.6005

**Figure 1:**
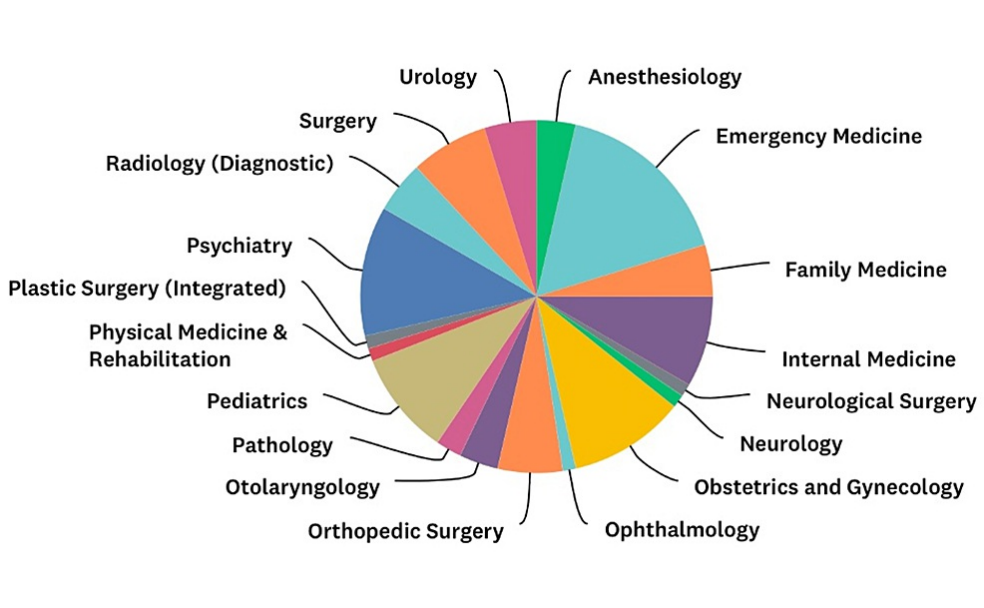
Diversity of residencies pursued by the class of 2022.

The self-assessment of knowledge, satisfaction, and experiences survey response rate for the 2022 graduating class was 84 of 99 (85%) students. Figure 1 demonstrates the diversity of residencies these students planned to pursue. Seventy-five students (89%) rated the PoCUS curriculum as superior or above average. Seventy-one of 84 (85%) anticipate using PoCUS after graduating from medical school. Sixty-four students (76%) agreed with the statement “there should be more point-of-care ultrasound incorporated in your medical education curriculum.”

**Figure 2:**
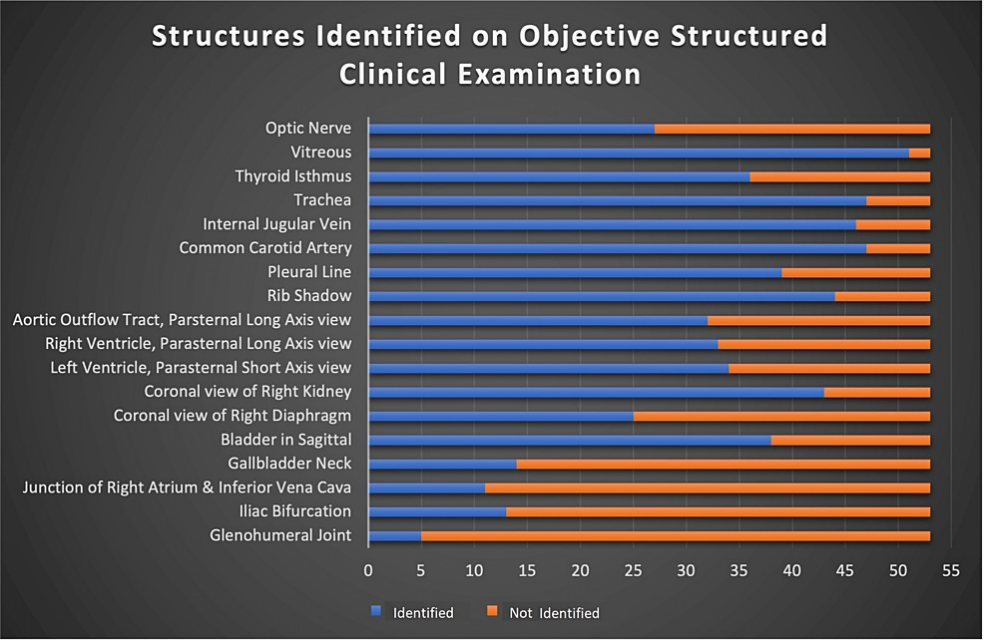
Objective structured clinical examination results.

OSCEs were administered to 53 of 65 eligible year four students (82%). Students were able to correctly identify a median of 11 (interquartile range {IQR}: 9.5-13) of 18 possible structures during these timed sessions or more than one structure per minute. Figure 2 demonstrates the success of identification according to structure.

## Discussion

Our institution created a sustainable and vertically integrated four-year PoCUS curriculum with improvements in student knowledge and skills that has been well received by students and has overcome limited faculty time and resources. Strategies including handheld ultrasound devices, near-peer teaching, flipped classroom formats and gamification, and staggered small group scheduling allowed us to prioritize hands-on practice and low student-to-instructor ratios.

Students demonstrated the most improvement on questions pertaining to fundamental PoCUS concepts such as orientation, transducer selection, and modes (i.e., questions 2, 3, 5, and 7 in Table 3) that are repeated across years in different contexts during the curriculum. Questions where there was little improvement had higher initial scores with anatomical foundations that may give first-year medical students an advantage.

The OSCE demonstrated students were able to independently acquire and identify more than one structure per minute using PoCUS. Given the high success of identifying structures at the top of the list and lower success of identifying structures at the end of the list, success was likely partially limited by the timed environment. Additionally, more superficial and prominent structures such as the trachea, vitreous, and neck vessels were likely easier to acquire without sophisticated transducer manipulation than deeper structures such as the heart and abdominal anatomy which can hide behind ribs and bowel gas, respectively. More confident students likely went faster and spent less time on each structure, allowing them to have time to identify structures near the end of the list.

Few medical schools have achieved a required four-year PoCUS curriculum with a comprehensive, multi-faceted approach [7,25]. And among those that have achieved a longitudinal PoCUS curriculum, none report knowledge and OSCE outcomes representative of all graduating students. For example, Bahner et al. reported OSCE performance among a select group of fourth-year medical students who applied and were accepted into an advanced ultrasound course [26]; and Hoppmann et al. reported excellent OSCE scores among first- and second-year students but did not repeat the assessment for graduating students [10], It is difficult to compare the knowledge and OSCE outcomes in our study to other’s work, as prior studies enrolled small samples of students, volunteers, covered fewer applications, or did not formally assess knowledge or OSCE scores [10,27,28]. However, most studies have found PoCUS curricula to acceptable to students, with some improvements in understanding of anatomy and physiology, physical examination skills, diagnostic skills, and confidence [3,6,28,29].

A 2021 review by Glass et al. of longitudinal PoCUS curricula reported that consistent ultrasound training and practice during the clinical years is uncommon in UME [25]. They note that medical schools with vertical PoCUS curricula tend to target clinical medical students interested in specific specialties where ultrasound is frequently used, such as radiology, emergency medicine, and obstetrics/gynecology. In contrast, our experience suggests that it is possible to successfully engage medical students using PoCUS across all years, regardless of their intended specialties.

Our findings are limited by the single site and relatively small medical school size, which may reduce generalizing to larger schools. The student surveys were developed by content experts and not tested, thus, respondents may not have interpreted questions as intended. Additionally, survey response rates were consistently more than half of the eligible students, but some were less than optimal. It is not yet clear that skills practiced during UME will translate to useful integration of PoCUS in clinical diagnosis and management during graduate medical education and beyond. In addition, our program evaluation did not formally include all stakeholders, such as other course directors and UME leaders. However, the program has been continuously supported and sustained for over six years in an overcrowded UME curriculum, with enthusiasm expressed informally by UME curriculum leadership.

Potential future research includes evaluating skills improvements by increasing inter-specialty collaboration, expanding hands-on skills practice during clinical clerkships, inserting more OSCEs at specific points in the curriculum, and adding more virtual teaching modules. Qualitative research methods to explore which elements are most effective for learning PoCUS would help to define the essential components of a longitudinal PoCUS curriculum. In addition, research examining program directors’ perceptions of resident PoCUS skills as well as residents’ perceptions of their UME PoCUS curriculum preparation will be important.

## Conclusions

This study describes a vertical PoCUS curriculum integrated across four years of UME and offers insight into both creating and incorporating a similar curriculum into a medical school with limited financial and faculty resources. The combination of near-peer teaching, low student-to-faculty ratio, and the incorporation of handheld ultrasound devices offered a unique educational experience for students. Students who completed this curriculum showed an increase in ultrasound knowledge and skill, and they rated the curriculum highly.

## Appendices

**Table 4:** Objective structured clinical examination. RA-IVC: right atrium-inferior vena cava

Please identify	Structure identified correctly	Structure not identified correctly
Optic nerve		
Vitreous		
Thyroid isthmus		
Trachea		
Internal jugular vein		
Common carotid artery		
Pleural line		
Rib shadows		
Parasternal long axis view, aortic outflow tract		
Parasternal long axis view, right ventricle		
Parasternal short axis view, left ventricle		
Coronal view of right kidney		
Coronal view of right diaphragm		
Bladder in sagittal		
Gallbladder neck in long axis		
RA-IVC (caval junction) in long axis		
Iliac bifurcation in short axis		
Glenohumeral joint		
